# Morpho-Agronomic Characterization of an Unexploited Germplasm Collection of Cauliflower (*Brassica oleracea* var. *botrytis* (L.)) from Spain

**DOI:** 10.3390/plants14182919

**Published:** 2025-09-19

**Authors:** Eric Prendes-Rodríguez, Alicia Iborra, Carla Guijarro-Real, Adrián Rodríguez-Burruezo, Ana Fita

**Affiliations:** 1 Instituto Universitario de Conservación y Mejora de la Agrodiversidad Valenciana (COMAV), Universitat Politècnica de València, Camino de Vera s/n, 46022 València, Spain; aibolop@posgrado.upv.es (A.I.); adrodbur@upvnet.upv.es (A.R.-B.); 2 Department of Biotechnology-Plant Biology, School of Agricultural, Food and Biosystems Engineering, Universidad Politécnica de Madrid, 28040 Madrid, Spain; carla.guijarro.real@upm.es

**Keywords:** landraces, phenotypic diversity, seed viability, earliness, curd architecture, multivariate analysis, genebank conservation, breeding targets

## Abstract

Cauliflower landraces (*Brassica oleracea* var. *botrytis*) safeguard allelic diversity for adaptation, yet their phenotypic breadth under winter field conditions remains under-documented. We evaluated 69 Spanish landraces and two commercial checks from the COMAV-UPV genebank using 15 quantitative and 21 qualitative descriptors. Seed viability ranged from 0 to 92%, and mature plants showed wide ranges in stem length (coefficient of variation ≈ 72%), leaf size, and head weight (100–723 g). Six curd-colour classes—including uncommon purple and Romanesco green—were recorded. Most accessions (>88%) required more than 120 days from sowing to harvest, but a distinct subset (12%) matured within 60–120 days. Plant stature tended to be positively associated with head mass, whereas highly branched inflorescences matured earlier. Variation was dominated by curd size and plant architecture. Multivariate analyses—principal component analysis for quantitative traits, multiple correspondence analysis for qualitative traits, factor analysis of mixed data, and clustering of FAMD scores by k-means—resolved three phenotypic clusters spanning a gradient of curd size/architecture and plant stature. The collection includes accessions with compact curds, earliness, or distinctive pigmentation that are immediately useful for breeding and for prioritizing regeneration. These results provide a phenotypic baseline for future genomic association studies and the development of cultivars adapted to winter production.

## 1. Introduction

*Brassica oleracea* embraces a remarkable array of vegetables—cauliflower, broccoli, cabbage, and kale—whose domestication history spans from the Eastern Mediterranean through classical Greece and Rome to most vegetable-growing regions worldwide [1]. Today Asia supplies ≈ 81% of global broccoli-plus-cauliflower output, whereas the Mediterranean basin accounts for 8%, with Spain, Italy, France and the United Kingdom as leading producers [2]. Despite broccoli dominating Spanish acreage, cauliflower (*B. oleracea* var. *botrytis*) retains strong agricultural and cultural importance in long-standing production areas [3].

In Mediterranean environments, characterized by recurrent drought and episodes of high temperature, cauliflower remains a key crop in local food systems. Advances in cultivation practices and breeding have improved yields and resilience in many locations [4,5,6,7], yet continued adaptation to site-specific agroclimates is needed to sustain production under ongoing climatic change.

In long-established production regions, local practices fostered the emergence of landraces adapted to farmers’ needs and to prevailing climates. Cauliflower landraces are agronomically valuable due to their evolved tolerance to biotic and abiotic stresses [8,9], and their conservation is intertwined with culinary traditions and regional identity [10,11,12,13]. Harnessing this diversity requires systematic characterization—morpho-agronomic, biochemical, and increasingly genomic. Broad surveys across crops have shown that combining qualitative and quantitative descriptors efficiently reveals useful variation in yield components, earliness, and quality traits [14,15,16,17,18,19,20]. Phenotypic diversity has been documented in *Brassica carinata* [21], *B. juncea* [22], and *B. napus* [23] and other *Brassica* collections [24,25,26,27]. However, Spanish cauliflower landraces remain under-characterized. Moreover, genebanks established to stem genetic erosion often retain only passport data and rudimentary descriptors [28,29], which hinders the triage of accessions for regeneration when seed viability declines and limits their immediate use in breeding. Deep phenotyping is therefore a prerequisite for exploiting accessions in breeding or for genome-wide association studies [30,31,32].

The Institute for the Conservation and Improvement of Valencian Agro-diversity (COMAV) at the Universitat Politècnica de València curates the largest Spanish working set of cauliflower germplasm—52 of the 72 national accessions deposited in the Centro Nacional de Recursos Fitogenéticos [33]. This specialization positions the COMAV genebank as a valuable resource for research and breeding programmes aimed at improving adaptability, yield, and quality. Yet its collection has never been comprehensively surveyed under field conditions typical of Mediterranean winter cropping. Here, we assess the morpho-agronomic diversity of 69 cauliflower landraces (plus two commercial cultivars) from the COMAV genebank grown under Mediterranean open-field conditions. We recorded 15 quantitative and 21 qualitative traits, applied multivariate and clustering analyses, and explored the relationship between plant architecture and curd development. Specifically, we (i) establish a detailed phenotypic baseline for this Spanish collection, (ii) identify landraces with compact curds, earliness, or distinctive pigmentation that are immediately useful for breeding and for prioritizing regeneration, and (iii) provide trait data to underpin forthcoming genomic association work and the development of resilient, high-value cultivars.

## 2. Results

### 2.1. Seed Viability and Adaptation to the Cultivation Conditions

**Figure 1:**
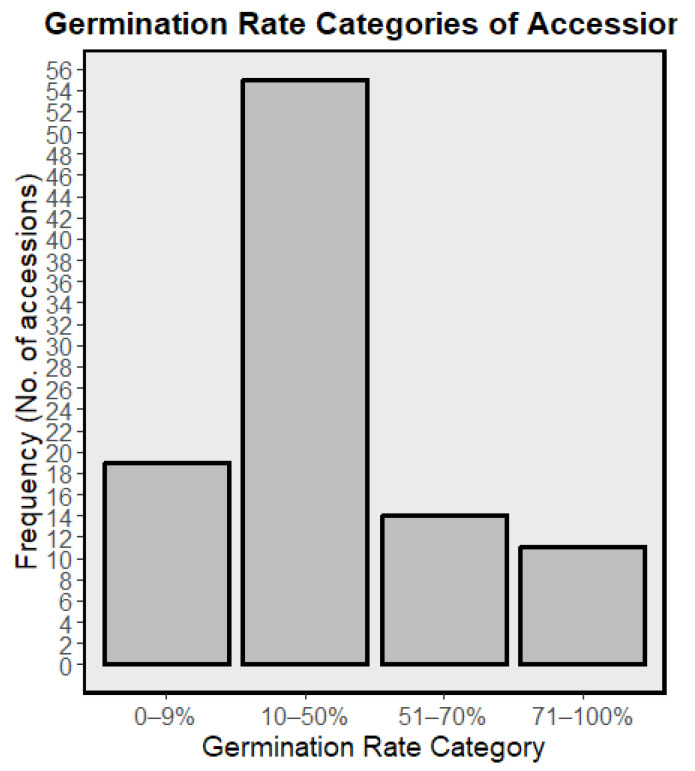
Histogram showing the distribution of germination rates (%) among the 99 accessions; bars indicate the number of accessions per viability class.

Eight days after sowing, germination of the 99 COMAV accessions ranged from 0 to 92.4% (mean ± SD = 34.9 ± 23.9%; SE = 2.4%; Figure 1). A total of 13 accessions exhibited very low viability (<10%), 51 fell in the low class (10–50%), 17 reached moderate levels (51–70%) and only 11 surpassed the ≥70% threshold generally considered acceptable for field trials, with 7 of those attaining 80–92% germination. A total of 7 accessions failed to emerge entirely and were discarded. Of the 92 lots that did germinate, just 69 landraces developed commercial heads under the trial conditions; together with the two F_1_ reference cultivars ‘Benidorm’ and ‘Amistad’, these entries constitute the phenotypic dataset analyzed in subsequent sections. The 23 accessions that germinated but never reached head maturity further reduced representation from several provinces (La Rioja, Cádiz, Girona, Barcelona, Tarragona, Castellón, Valencia, Alicante, Murcia, Zaragoza, Huesca and Cáceres).

### 2.2. Phenotypic Diversity in the COMAV Genebank Collection

#### 2.2.1. Quantitative Descriptors

Fifteen quantitative traits were measured in the 69 landraces that reached commercial maturity (Table 1). Relative dispersion was greatest for stem length below the floral head (CV ≈ 72%), followed by petiole length (≈49%), leaf width (≈33%) and petiole width (≈33%). Stem length varied ten-fold, from 1.43 to 14.25 cm (mean ± SE = 4.11 ± 0.35 cm), whereas petiole length ranged from 1.00 to 3.20 cm (1.23 ± 0.07 cm) and leaf width from 10.50 to 49.20 cm (22.36 ± 0.87 cm). Head weight showed the widest absolute range (100–723 g) and a coefficient of variation of ~37% (359 ± 15.5 g).

By contrast, variables describing overall plant stature were comparatively uniform (CV < 25%). Plant height spanned 32.0–77.6 cm (54.23 ± 1.22 cm; CV ≈ 19%), and plant width 40.0–121.2 cm (81.78 ± 1.99 cm; CV ≈ 21%). Head length (4.20–13.33 cm, 8.20 ± 0.24 cm) and head diameter (5.10–17.30 cm, 10.91 ± 0.31 cm) displayed moderate dispersion (both CV ≈ 25%). The number of leaves and/or scars was the most homogeneous trait (14–26, mean = 20.01 ± 0.38; CV ≈ 16%).

#### 2.2.2. Qualitative Descriptors

**Figure 2:**
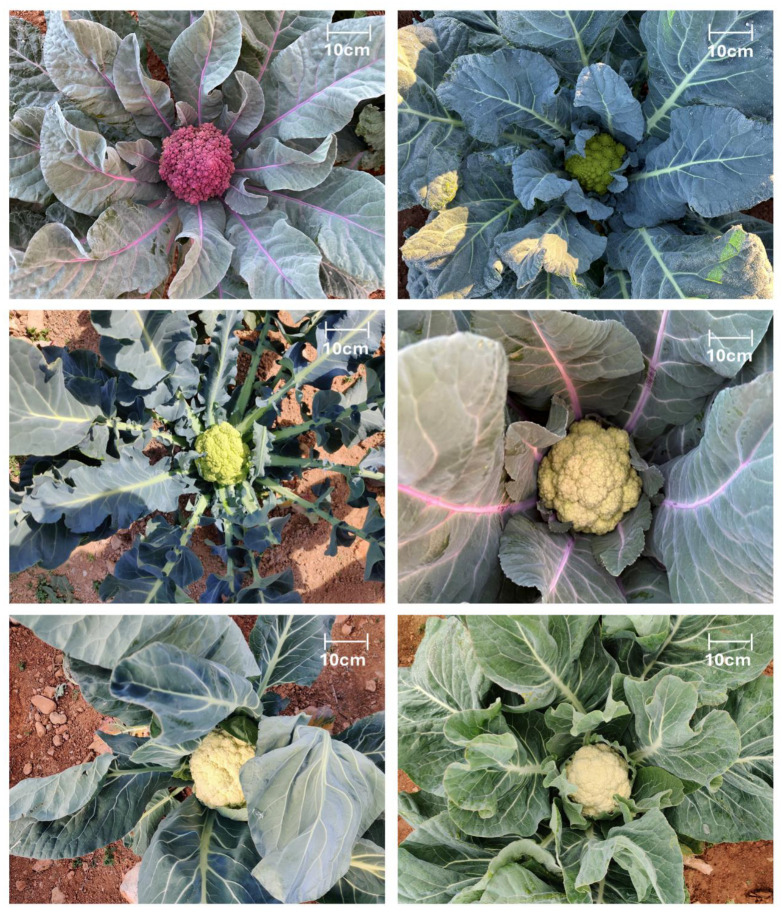
Representative examples of morphological diversity in the COMAV cauliflower collection. The six photographs illustrate variation in plant architecture, leaf colour, lobing, blistering, leaf contour, petiole pigmentation, and curd colour/structure (white, green-Romanesco, yellow-green and purple). All pictures were taken in the field with a smartphone mounted on a tripod 1.5 m above the plant; scale bar = 10 cm.

Most landraces showed an erect plant habit (68%, 47/69), with the remainder semi-erect (Table 2). Leaf blades were predominantly elliptic (55%), and sinuate (55%) or lyrate (30%) lobing was common; lanceolate (16%), spathulate (12%) and obovate (10%) outlines occurred at lower frequencies. Leaf apices were chiefly intermediate (64%) or broadly rounded (25%), while blistering was low in three-quarters of the accessions and absent or intermediate in the rest. Foliage colour ranged from light-to-dark-green in 95% of the material, with purple-green blades recorded only sporadically (4%; Figure 2). Leaf lobing was absent in 81% of accessions. Note that LEAFLOB is a binary score for deep lobing; many accessions scored ‘sinuate’ or ‘lyrate’ on LDIV without meeting the threshold for deep lobing, hence high ‘absent’ in LEAFLOB can co-occur with sinuate/lyrate LDIV. Petioles were mainly semi-round (57%) and light-green (84%).

**Figure 3:**
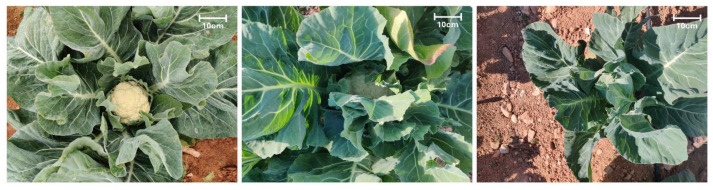
Head exposure by outer leaves (HCOV). Representative examples of the three categories scored at commercial maturity. From left to right: exposed, intermediate, and covered. All photos taken in the field; scale bar = 10 cm.

For curd traits, 98% of accessions formed well-defined heads. Heads were typically exposed (57%) or partly covered (41%) by outer leaves (Figure 3). Longitudinal profiles were concave (55%) or flat (32%), seldom spherical (14%). Stem estimates were short to intermediate in more than 90% of entries. Solidity was intermediate (51%) or high (47%), and splitting tendency was low to intermediate in every case. The prevailing branching pattern combined a terminal head with smaller auxiliary shoots (62%); compact and loosely branched types each accounted for 18%. Medium head size predominated (62%), and the head-to-plant ratio was small in 29% of landraces.

**Figure 4:**
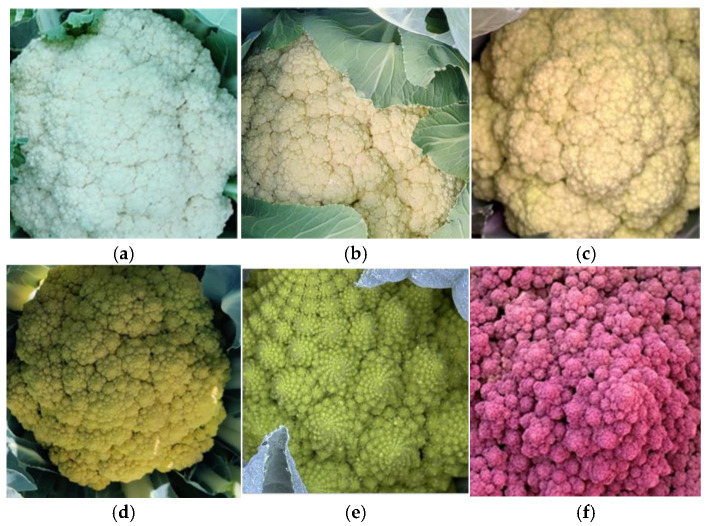
Close-up images illustrate the main colour classes recorded were white (**a**) and different shades of cream (**b**,**c**), with lower frequencies of yellow (**d**), green (**e**), and purple (**f**). The images also highlight differences in head solidity, where more compact heads appear denser, while looser heads display a more open structure.

Six curd-colour classes were recorded (Figure 4). White heads were most frequent (55%), followed by yellow-green (26%) and cream (11%); yellow, green, and purple heads each occurred in 2–4% of accessions. The purple-headed landraces BGV001728, BGV001744 and BGV001747, and the Romanesco green BGV004798, illustrate the extremes of colour diversity (Figure 4e). In terms of earliness, 88% of accessions required >120 days from sowing to harvest under Mediterranean winter conditions, whereas eight entries (12%) matured within 60–120 days—seven from Catalonia and one from the Valencian Region.

### 2.3. Associations Among Morpho-Agronomic Traits

**Figure 5:**
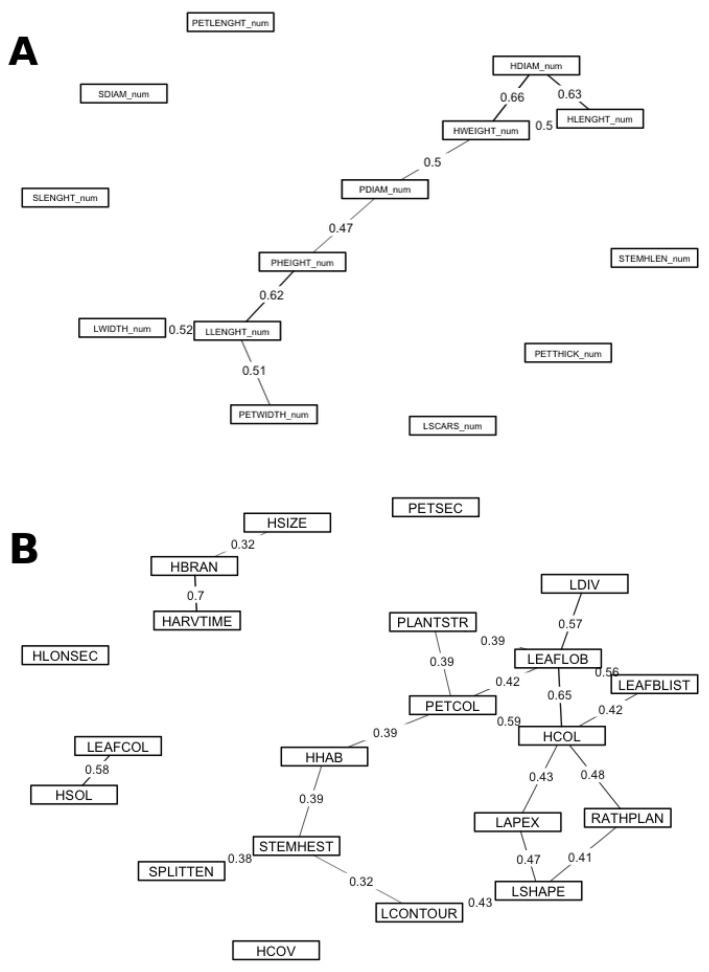
Trait association networks. (**A**) Quantitative traits: Spearman’s rank correlation among 15 quantitative descriptors; edges are shown for |ρ| ≥ 0.40 after Benjamini–Hochberg false discovery rate adjustment (FDR ≤ 0.05). Line width scales with |ρ|; solid lines indicate positive associations and dashed lines indicate negative associations. (**B**) Qualitative traits: Cramér’s V among 21 qualitative descriptors; edges are shown for V ≥ 0.30 with FDR ≤ 0.05; line width scales with V. Node labels follow the descriptor acronyms defined in the Materials and MethodsNo mixed (quantitative–qualitative) correlations are reported.

We analyzed associations separately for quantitative and qualitative variables to avoid mixing scales. For quantitative traits, several moderate-to-strong Spearman correlations (|ρ| ≥ 0.50; *p*-values adjusted using the Benjamini–Hochberg false discovery rate, FDR ≤ 0.05) involved curd size and plant architecture (Figure 5A). Head weight correlated with head diameter (ρ ≈ 0.66) and head length (ρ ≈ 0.50), and also with plant width (ρ ≈ 0.50). Head diameter and head length were tightly linked (ρ ≈ 0.63). Plant height was positively related to plant width (ρ ≈ 0.47) and leaf length (ρ ≈ 0.62), reflecting overall vegetative vigour. Leaf length covaried with leaf width and petiole width (both ρ ≈ 0.51), and petiole thickness correlated with petiole width (ρ ≈ 0.42) and head weight (ρ ≈ 0.44).

For qualitative traits, Cramér’s V revealed moderate-to-strong associations (V ≥ 0.30; FDR ≤ 0.05) (Figure 5B). The branching pattern of the inflorescence was strongly associated with time to harvest (V ≈ 0.70): highly branched curds tended to mature earlier than compact-headed types. Leaf lobing was associated with the degree of leaf division (V ≈ 0.57) and with head-colour class (V ≈ 0.65). Consistent colour cues emerged across organs: petiole/midrib colour was associated with head colour (V ≈ 0.59). Additional associations of moderate magnitude involved leaf blistering with leaf lobing (V ≈ 0.56), the head-to-plant size ratio with head colour (V ≈ 0.48), and leaf apex with leaf blade shape (V ≈ 0.47).

Taken together, these patterns indicate that curd size covaries with plant stature and leaf dimensions, whereas categorical traits group into colour–morphology syndromes and an earliness–branching axis.

### 2.4. Principal-Component and Multiple Correspondence Analyses

**Figure 6:**
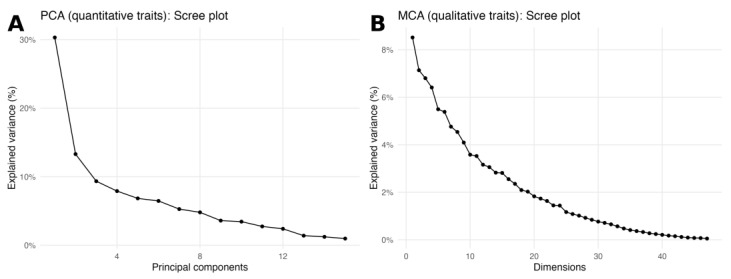
Scree plots for block-wise ordinations. (**A**) PCA scree plot for the 15 quantitative traits; the curve shows the percentage of variance explained by each principal component. (**B**) MCA scree plot for the 21 qualitative traits; the curve shows the percentage of variance explained by each dimension. Both panels display a gradual eigenvalue decay with no single dominant axis.

To explore the two data blocks independently, a PCA was run on the 15 quantitative descriptors and an MCA on the 21 qualitative descriptors. Both scree plots showed a gradual decay of eigenvalues without a single dominant axis (Figure 6A,B). In the PCA, the first components were driven by curd size (head diameter, head length, head weight) together with plant architecture (plant width and leaf dimensions). In the MCA, the leading dimensions were mostly shaped by leaf lobing/division, head and petiole colour, and head solidity. These block-wise patterns are consistent with the association networks in Figure 5.

### 2.5. Multidimensional Analysis

**Figure 7:**
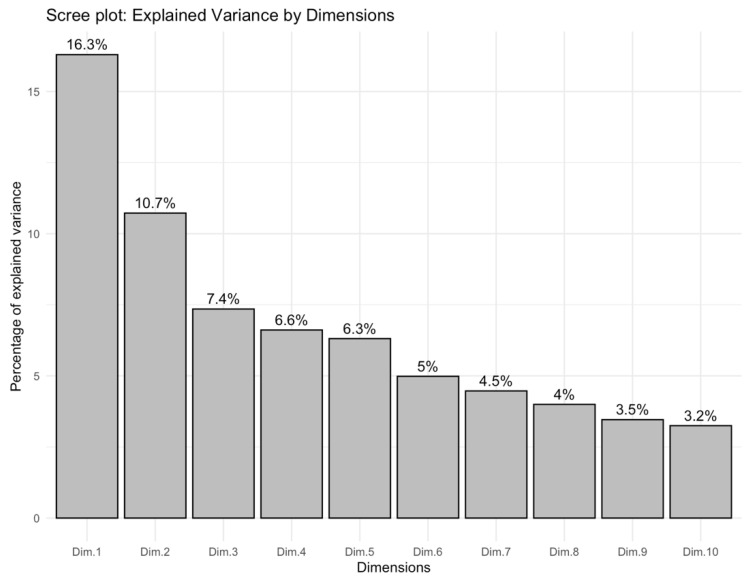
Scree plot of the percentage of variance explained by the first ten FAMD dimensions.

A factorial analysis of mixed data was performed on the full descriptor set (21 qualitative + 15 quantitative traits). The scree plot (Figure 7) shows that Dim-1 and Dim-2 explain 16.3% and 10.7% of the total variance, respectively. The first five dimensions jointly captured 47.3%**,** while the first ten dimensions together accounted for 67.5% of the inertia. Because no single dimension dominated—and the eigenvalue curve displayed a clear elbow after Dim-10—subsequent variable-importance calculations and clustering procedures were based on the ten-dimension solution.

#### 2.5.1. Variable Contribution

Weighted, normalized contributions across the first ten FAMD dimensions (Table 3) were led by colour and shape descriptors: head surface colour (HCOL, 4.15%), leaf shape (LSHAPE, 4.02%), and the longitudinal profile of the curd (HLONSEC, 3.05%). Additional influential traits included leaf blistering (LEAFBLIST, 2.66%), leaf colour (LEAFCOL, 2.56%), leaf apex shape (LAPEX, 2.28%), petiole/midrib colour (PETCOL, 2.15%), the head/plant size ratio (RATHPLAN, 2.08%), head stem estimation (STEMHEST, 1.97%), and leaf division (LDIV, 1.91%). Overall, colour/shape descriptors and plant stature-related features make the largest contributions to the multivariate structure of the collection.

#### 2.5.2. Cluster Analysis

**Figure 8:**
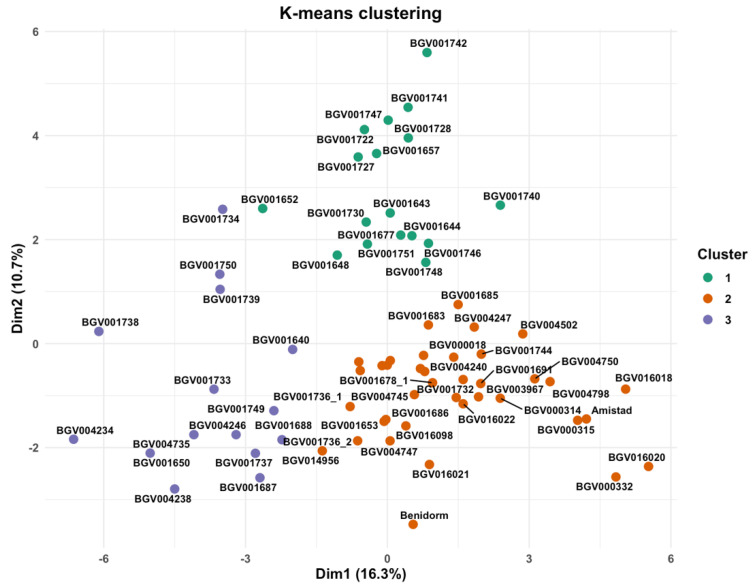
Structure of the collection in the FAMD space. Scatter of individuals on Dim-1 vs. Dim-2 coloured by k-means clusters (k = 3) computed from the first ten FAMD dimensions (average silhouette ≈ 0.17). Dim-1 = 16.3%, Dim-2 = 10.7%.

The k-means partition of the FAMD scores (first ten dimensions) yielded three groups (k = 3; average silhouette ≈ 0.17), indicating modest separation with partial overlap in the Dim-1/Dim-2 projection (Figure 8). The three clusters mainly split the collection along curd size/architecture and earliness/colour axes, consistent with the association networks and block-wise ordinations. Although the partition was obtained with ten axes, the two-dimensional scatterplot already shows partial separation with overlap among the three groups, consistent with the k-means solution from the full multidimensional space.

**Figure A1:**
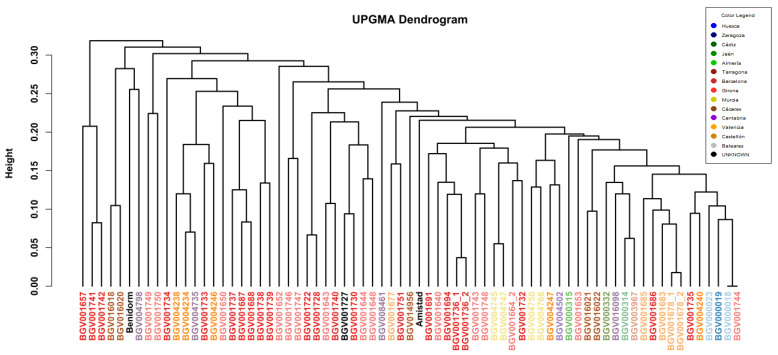
UPGMA dendrogram from Gower distances. Relationships among 69 cauliflower accessions computed from the full set of 36 descriptors (21 qualitative + 15 quantitative). The dendrogram preserves ≈75% of the original pairwise dissimilarities (cophenetic r = 0.751) and recovers regional affinities, consistent with the FAMD-based clustering in the main text.

As an exploratory cross-check, a Gower–UPGMA dendrogram shows consistent affinities (cophenetic r = 0.751); see Appendix A Figure A1.

## 3. Discussion

Our results document substantial heterogeneity in seed viability and broad morphological diversity within the COMAV cauliflower collection grown under Mediterranean winter conditions. Most accessions showed low-to-moderate germination, only a subset reached market maturity, and phenotypic structure was chiefly driven by curd size/architecture and plant stature, with colour and leaf form providing secondary axes. Treating the two data blocks separately (PCA for quantitative traits; MCA for qualitative traits) and jointly (FAMD) delivered a consistent picture and supported a three-group partition with modest but non-random separation.

### 3.1. Germination and Growth Performance

Seed viability across the 69 cauliflower accessions averaged 35% (range 4–88%), and only 11 accessions retained ≥80% germination. Such heterogeneity mirrors the marked viability decline reported for long-stored cereal and maize collections and emphasizes the need for stringent seed-quality monitoring in ex situ programmes [34,35,36].

A probable cause for this is that most lots were harvested 30–40 years ago and have never been regenerated. Seed aging is driven by progressive oxidative stress that disrupts membranes and macromolecules, ultimately curbing germination [37,38]. Without periodic regeneration, genetic erosion becomes inevitable as non-viable samples drop from the collection. Also, Brassica seeds are not exceptionally long-lived under genebank conditions. Comparative studies show appreciable viability loss after 20–30 years even at −18 °C [38,39]. Empirical work on *Brassica* and allied taxa recommends regeneration every 10–20 years to keep viability above 70% [40,41,42,43]. Our data confirm that most accessions have now reached or exceeded that window, making regeneration an urgent priority.

Germination tests identify which accessions are most at risk; those below 50% viability should be scheduled for regeneration in the next planting season, while lots of accessions between 50 and 75% warrant close monitoring. Stratifying regeneration effort in this way minimizes further viability loss and safeguards unique alleles [44].

The failure of accessions to reach commercial maturity from diverse regions suggests that both environmental and genetic factors influence plant growth and development. The presence of failed accessions from various provinces indicates that localized environmental conditions, including soil type, temperature fluctuations, and water availability, may significantly impact seedling survival and establishment. Studies have shown that seedling development and establishment are influenced by additional environmental stressors, including temperature extremes, light availability, water potential, and nutrient levels. Insufficient water or suboptimal nutrient availability during early seedling growth can lead to stunted development, reduced vigour, and ultimately poor crop establishment, limiting the chances of accessions reaching commercial maturity. Furthermore, the physical properties of soil, such as texture and structure, directly impact root penetration and seedling anchorage, further affecting plant development [45,46,47].

These findings underscore the necessity of accounting for regional environmental conditions when cultivating and evaluating germplasm collections, as environmental mismatches can significantly hinder plant establishment. For instance, local environmental conditions and grazing pressure have been shown to influence species performance and affect spatial patterns of genetic diversity in contrasting ways. Developing adaptive cultivation strategies that consider these local environmental factors can help mitigate challenges and improve the overall performance of accessions in field trials [48,49,50].

Because we used an augmented randomized design with unequal plants per accession and no plot replication for test entries, all inferences are descriptive at the accession level. Commercial checks replicated by blocks were used to monitor field heterogeneity, and association tests were controlled for multiple comparisons (Benjamini–Hochberg FDR). These design choices, together with separate PCA/MCA and joint FAMD analyses, aim to maximize transparency while avoiding over-interpretation.

### 3.2. Phenotypic Diversity and Multidimensional Analysis

Analyzing the two data blocks independently supported these same axes of variation. In the PCA, the leading components were dominated by curd size (head diameter and length, head weight) plus plant width and leaf dimensions; in the MCA, the first dimensions were shaped by leaf lobing/division, head and petiole colour, and head solidity. Both scree plots displayed gradual eigenvalue decay with no single dominant axis, matching the association networks and the joint FAMD structure.

The COMAV collection exhibited a breadth of vegetative and curd variation that rivals the ranges reported for global cauliflower panels. Quantitative traits spanned one (number of leaves) to ten-fold (stem length below the head) differences among accessions, while qualitative characters such as leaf division, blistering, and head colour filled virtually every class listed in the Descriptors for *Brassica* and *Raphanus* [51]. Such dispersion attests to long-term farmer selection under contrasting Mediterranean micro-climates rather than recent, uniform breeding criteria.

The UPGMA dendrogram, generated using Gower’s distance, provides meaningful insights into this diversity. Accessions grouped together share similarities in key traits such as head size, head colour, and secondary morphological features, indicating genetic or phenotypic proximity. For instance, a cluster including accessions such as BGV001657, BGV001741, and BGV001742 comprises genotypes with small to medium head sizes (200–400 g) and uniformity in leaf traits, with moderate leaf lobation and blistering. Conversely, another cluster containing accessions like Benidorm, BGV004798, and BGV001750 includes genotypes with larger heads (>500 g) and broader plant architecture. Consistent with this, the FAMD + k-means partition retrieved similar affinities along curd size/architecture and leaf form, so we interpret UPGMA as a complementary visualization rather than a primary classifier.

Head colour contributed to some sub-groupings—greenish heads predominated in the former domain—yet plant stature and curd mass were the stronger discriminants. A comparable dominance of size-related descriptors over pigmentation was observed in a 92-genotype Indian panel where clustering aligned with curd weight, plant architecture, and leaf length rather than with geographic provenance [52]. Similarly, ref. [53] demonstrated that genetic diversity in cauliflower inbred lines was associated with phenotypic traits, with major differentiation occurring in head size and leaf morphology rather than solely through molecular markers. Likewise, in broccoli, the major split between landraces and modern hybrids hinged on head diameter and compactness, not on secondary traits [54].

Recent genomic studies lend mechanistic support to these morphological patterns. A chromosome-scale survey of 971 cauliflower genomes showed that stepwise domestication from leafy progenitors to compact curd formation was driven by structural variants at MADS-box loci (*BoCAL*, *BoFUL*, *BoAP1*) and by a zinc-finger regulator of stem height [55]. The clear separation between compact-headed and highly branched accessions in our dendrogram is therefore likely to mirror allele segregation at those loci within Spanish landraces. In line with this, we identified several accessions that consistently produced compact curds, confirming one of our main objectives and underscoring their breeding relevance.

Leaf architecture emerged as an almost equally powerful axis of diversity: leaf shape ranked second in FAMD importance (4.1% of explained variance). Variation in the promoter of the *BoLMI1a* gene, recently mapped in ornamental kale, underlies lobed versus entire leaf margins and has been linked to heat dissipation and high-density planting potential [56]. The coexistence of elliptic, lobed, and lanceolate blades in our panel suggests that such regulatory polymorphisms are also segregating in traditional cauliflower. Head longitudinal profile (concave–flat) was the third most influential descriptor. Concave or flat curds satisfy wholesale standards for compactness, whereas looser profiles may enhance aeration and reduce fungal incidence under the humid Mediterranean winters; the near-even split (55% concave, 32% flat) indicates parallel selection for both market channels. Similar duality was recorded in multi-environment trials, where compactness correlated positively with yield but inversely with tolerance to autumn rainfall events [54].

The three-group partition derived from the first ten FAMD axes (Figure 7) is statistically admissible but biologically nuanced. A mean silhouette width of 0.17 indicates modest cohesion and separation—typical when clustering landraces that have been subjected to overlapping farmer-selection criteria—yet values above zero still reflect structure beyond random allocation [57]. Cluster 1 (17 accessions, green symbols) occupies the positive sector of both Dim1 (16.3%) and Dim2 (10.7%), dimensions dominated by head size, compact longitudinal profile and elliptic leaves. Members of this group, including BGV001742, BGV001741, and BGV001747, show the heaviest curds (>500 g) and the highest solidity scores, suggesting convergent selection for marketable yield and transport resilience. Cluster 3 (15 accessions, purple) is confined to the negative tail of Dim1 and contains lines such as BGV004234 and BGV001738 that combine small heads, slender stems, and reduced plant spread, a phenotype historically preferred for low-input or closely spaced cultivation systems. The bulk of the collection (cluster 2, 38 accessions, orange) forms a broad centroid around the origin, representing intermediate morphotypes that bridge the two extremes. This continuum agrees with earlier multivariate surveys in cauliflower where head-weight classes, rather than strict geographic origin, produced the primary stratification of diversity [58,59,60].

Taken together, head architecture, plant stature, and leaf morphology are the main factors explaining the principal structure of phenotypic diversity in the COMAV cauliflower collection, concordant with previous morphological surveys and with current genomic dissection of curd biogenesis. The simultaneous presence of early and late, compact and branched, and chromatically diverse phenotypes within a single regional pool underscores the breeding value of Spanish landraces for yield improvement, climate resilience, and market differentiation.

### 3.3. Correlation Amongst Descriptors

We analyzed correlations within data type—Spearman’s rank correlations (ρ) for the 15 quantitative traits and Cramér’s V for the 21 qualitative traits—while mixed (quantitative–qualitative) relations were examined in the multivariate analyses. *p*-values were adjusted with the Benjamini–Hochberg false discovery rate (FDR ≤ 0.05).

For quantitative traits, several strong Spearman correlations (|ρ| ≥ 0.40; FDR ≤ 0.05) defined coherent modules of curd size and vegetative architecture (Figure 5A). The strongest links were head weight with head diameter (HWEIGHT_num–HDIAM_num, ρ ≈ 0.66), head diameter with head length (HDIAM_num–HLENGHT_num, ρ ≈ 0.63), and leaf length with plant height (LLENGHT_num–PHEIGHT_num, ρ ≈ 0.62). Head weight also scaled with head length (HWEIGHT_num–HLENGHT_num, ρ ≈ 0.50) and plant width (HWEIGHT_num–PDIAM_num, ρ ≈ 0.50). Plant size covaried internally (PHEIGHT_num–PDIAM_num, ρ ≈ 0.47). Leaf length formed a vegetative dimension with leaf width (LLENGHT_num–LWIDTH_num, ρ ≈ 0.52) and petiole width (LLENGHT_num–PETWIDTH_num, ρ ≈ 0.51). Petiole thickness associated with petiole width (PETTHICK_num–PETWIDTH_num, ρ ≈ 0.42) and with head weight (PETTHICK_num–HWEIGHT_num, ρ ≈ 0.44). These patterns agree with previous reports that larger plants tend to develop broader foliage and heavier curds [61,62,63,64,65,66,67,68].

For qualitative traits, Cramér’s V highlighted interpretable associations (Figure 5B). Inflorescence branching was strongly associated with earliness class (HBRAN–HARVTIME, V ≈ 0.70); contingency counts showed highly branched entries over-represented among earlier harvest classes. Leaf lobing connected with the degree of leaf division (LEAFLOB–LDIV, V ≈ 0.57) and with head-colour class (LEAFLOB–HCOL, V ≈ 0.65). Consistent colour cues emerged across organs: petiole/midrib colour tracked head colour (PETCOL–HCOL, V ≈ 0.59). Additional moderate links included leaf blistering with leaf lobing (LEAFBLIST–LEAFLOB, V ≈ 0.56), the head/plant size ratio with head colour (RATHPLAN–HCOL, V ≈ 0.48), and leaf apex with leaf shape (LAPEX–LSHAPE, V ≈ 0.47). (Cramér’s V measures strength but not direction; direction was inferred from contingency tables). These patterns are consistent with developmental genetics: LMI1-like regulators control leaf lobing in *B. oleracea*, MYB anthocyanin activators drive coordinated pigmentation across organs (explaining the PETCOL–HCOL link), and changes in inflorescence identity/timing that produce Romanesco-type branching illustrate how architecture can couple with maturity—providing a mechanistic basis for the strong HBRAN–HARVTIME association [69,70,71].

From a breeding perspective, these results point to a curd-size/plant-stature axis in the quantitative block and colour–morphology and earliness–branching axes in the qualitative block, consistent with the block-wise PCA/MCA and the joint FAMD structure. The earliness–branching relation echoes findings in *B. oleracea* crops where inflorescence architecture affects developmental timing and uniformity [54,72,73].

Spanish cauliflower landraces conserved at COMAV harbour immediately usable variation for breeding and conservation triage. For yield and market quality, accessions combining compact curds and high solidity coincide with larger plant stature and leaf size; for calendar management, highly branched curds tend to mature earlier; and for product differentiation, purple and Romanesco green heads provide rare pigmentation. Importantly, the compact-head accessions identified in this study represent particularly valuable materials for breeding programmes aimed at improving head solidity and fresh-market quality. Given the current distribution of seed viability, regeneration should prioritize low-viability but phenotypically distinctive entries to avoid losing unique combinations of curd architecture, earliness, and colour.

## 4. Materials and Methods

### 4.1. Plant Material

**Figure 9:**
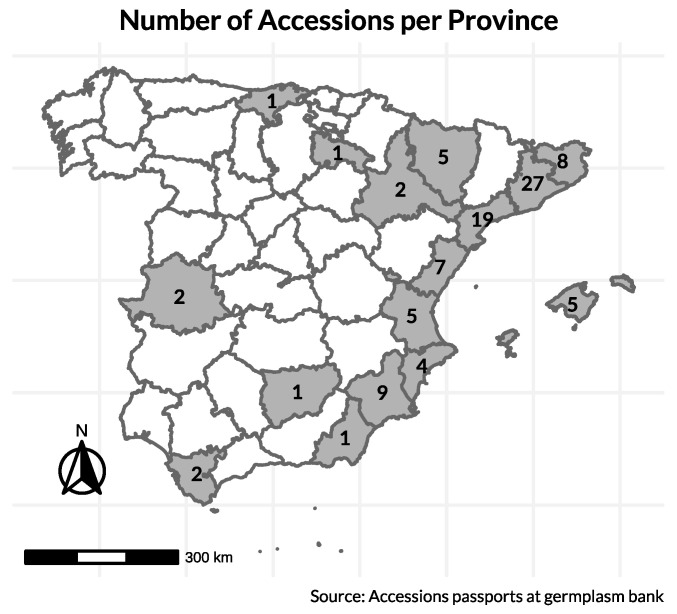
Geographic origin of the landrace accessions stored in the COMAV genebank and later sown; numbers indicate accessions per province. Commercial checks (‘Benidorm’, ‘Amistad’) are not included in province counts.

Initially, ~250 seeds per accession from a total of 99 accessions of *B. oleracea* were obtained from the COMAV genebank. These materials included 98 Spanish cauliflower landraces and 1 Romanesco-type landrace, originating from sixteen provinces (see map in Figure 9). Collection sites were defined from passport data registered at the COMAV genebank. In total, the accessions originally represented sixteen Spanish provinces at altitudes ranging from 6 m to 950 m above sea level. Additionally, two commercial cauliflower cultivars, ‘Benidorm’ and ‘Amistad’, were characterized as checks because they are widely grown and high-yielding under Mediterranean greenhouse and open-field conditions; they provide practical references for productivity, head quality, and earliness.

Because germination and emergence varied among accessions, only 75 accessions produced at least one vigorous plant and 69 reached commercial head stage. All analyses in this study are performed at the accession level: quantitative traits were averaged across plants within an accession, and qualitative traits were summarized as the modal category while recording any within-accession polymorphism.

### 4.2. Location and Conditions of Cultivation

The field trial was placed in Cheste, Valencia (39°29′38.8″ N 0°41′32.6″ W), during the 2022–2023 season. The electrical conductivity of the soil ranged from 0.82 to 1.21 dS/m throughout the cultivation cycle. The average daily temperatures varied between 3 and 20 °C, thus the plants accumulated about 600 chill hours, according to meteorological station reports [74]. Climate records from the IVIA-Cheste Agrometeorological Station (http://riegos.ivia.es/, accessed on 31 May 2025) indicate a mean annual rainfall of 430 mm, concentrated in autumn and spring. Drip irrigation was scheduled to maintain the soil at 70% of field capacity, delivering 250 mm of water during the cycle. Fertigation supplied a total of 180-60-240 kg ha^−1^ of N-P_2_O_5_-K_2_O (split in eight weekly applications) following regional cauliflower guidelines [75].

Seeds were germinated on the first week of August 2022 by direct sown on seedling trays using commercially available substrate, at the professional nursery *Planters Peris* (Alginet, Valencia). The germination rate was noted 8 days after sowing. Following germination, seedlings were selected and transplanted to the field trial, ensuring the inclusion of healthy and vigorous plants. Transplanting was conducted at the 4th leaf stage, except for accessions that failed to germinate.

Since germination and emergence were highly variable; consequently, only 75 accessions produced at least one vigorous plant, and 69 reached commercial head stage. All subsequent analyses are based on the plants that completed the cycle. Across the 69 accessions that produced a marketable head, the number of evaluable plants per entry ranged from 3 to 15.

The trial followed Federer’s augmented randomized design [76] to accommodate uneven plant numbers per accession. Each accession constituted a single, non-replicated plot of 1–15 plants, whereas the commercial checks were replicated in every block and used to monitor field heterogeneity. Given the lack of plot replication for accessions, we did not attempt variance partitioning into genetic and environmental components; results are therefore descriptive/exploratory at the accession level.

### 4.3. Morphological Variables

Morphological characterization followed the “Descriptors for *Brassica* and *Raphanus*” [51], with minor adaptations. A set of 36 descriptors was evaluated—15 quantitative and 21 qualitative (Table 4)—spanning plant (5), leaf (15), and head (16) traits. The final list prioritized (i) traits showing wide phenotypic variation in preliminary scouting and (ii) attributes valued by growers and the fresh market (e.g., head colour, solidity, earliness, leaf attitude).

Vegetative measurements were taken when ≥50% of plants within an accession had reached the flowering stage, whereas head-related traits were scored at commercial maturity (≥10 cm head diameter, compact surface, and typical colour for the accession without visible rice-like texture). For quantitative plant-level descriptors (e.g., stem length, plant width), all available plants were measured, and accession means were computed. Qualitative variables were scored on all plants and summarized as the modal category; within-accession polymorphism was recorded. For each quantitative descriptor we report the accession mean ± standard error (SE) to indicate precision under unequal replication.

### 4.4. Data Analysis

A database including both quantitative and qualitative variables was assembled at the accession level (means for quantitative traits; modal categories for qualitative traits). For quantitative descriptors, we computed summary statistics (mean, coefficient of variation, minimum, and maximum). For qualitative descriptors, we summarized category frequencies.

#### 4.4.1. Association Among Traits

Associations between quantitative traits were assessed with Spearman’s rank correlation (ρ). Associations between qualitative traits were evaluated with Cramér’s V (χ^2^-based). We did not compute simple correlations mixing categorical with numerical variables; instead, relationships between variables of different types were explored within the multivariate analyses described below. To account for multiple testing, *p*-values were adjusted using the Benjamini–Hochberg false discovery rate (FDR) procedure, and associations were considered significant at FDR ≤ 0.05.

#### 4.4.2. Multivariate Analyses by Data Type

(i) PCA was applied to the quantitative traits after centering and scaling to unit variance (z-scores). We computed the proportion of variance explained and the trait loadings on the leading components.
(ii) MCA was applied to the qualitative traits treated as factors (ordinal scales kept as ordered factors without numeric recoding). We computed the contributions of each category to the leading dimensions.
(iii) FAMD was used as an integrative analysis of the mixed dataset (quantitative + qualitative) to visualize joint structure. No numeric recoding was required because FAMD natively handles mixed tables. Global trait contributions were computed as variance-weighted averages over the first ten dimensions and then normalized to sum to 100% across variables.

#### 4.4.3. Clustering

Individual coordinates from the FAMD (first ten dimensions) were partitioned with k-means. We explored k = 2–6 and used the average silhouette width to guide the choice of k, complemented by interpretability of the partitions. The final solution retained k = 3. We ran k-means with multiple random starts to ensure stability (nstart = 500).

#### 4.4.4. Sensitivity Analysis

As a robustness check, we computed Gower’s distance and generated a UPGMA dendrogram, assessing cophenetic correlation to appraise tree faithfulness [77,78]. For this alternative distance-based analysis, ordinal qualitative traits were temporarily coded as consecutive integers, following common practice [77,78,79,80]. These results are provided in Appendix A.

All analyses were conducted in R version 4.3.1 [81]. PCA/MCA/FAMD and clustering were implemented with standard R packages.

## 5. Conclusions

Seed viability across the COMAV cauliflower collection is highly heterogeneous. Only one-tenth of the accessions retained ≥80% germination, and almost one quarter failed to produce commercial heads under Mediterranean winter conditions. These figures confirm that a substantial proportion of Spanish landraces now require urgent regeneration if their genetic integrity is to be preserved.

Despite the viability bottleneck, the 69 landraces that reached maturity encompassed striking morphological breadth. Quantitative descriptors ranged from ten-fold (stem length) to three-fold (head diameter) variation, while qualitative traits captured the full spectrum of leaf architecture, curd colour, and solidity recognized for the crop. Multivariate and clustering analyses converged on a simple organizing principle: curd-size attributes, together with overall plant stature and leaf shape, explain most of the phenotypic structure, whereas geographic origin plays a secondary role. Three FAMD-based clusters illustrate a continuum from small, slender plants with loose curds to large, wide-rosetted types bearing dense heads, with numerous intermediates bridging both extremes.

Trait correlations revealed predictable but useful linkages—larger plants invariably bore heavier heads—alongside a trade-off between earliness and curd compactness mediated by branching intensity. These relationships provide breeders with clear targets: early, highly branched forms for staggered production cycles, or compact, large-curd genotypes for fresh-market yield. Importantly, several accessions were identified that consistently produced compact curds, confirming one of the main objectives of this study. These genotypes represent valuable candidates for breeding programmes seeking to improve head solidity and marketable quality under Mediterranean conditions.

In sum, the COMAV collection retains ample morphological and agronomic diversity even after decades of ex situ storage. Prioritized seed regeneration, coupled with trait-informed selection, will allow this diversity to underpin breeding programs aimed at yield improvement, climate resilience, and niche-market differentiation.

## Figures and Tables

**Table 1:** Mean ± standard error (SE), coefficient of variation (CV%), and range of 15 quantitative traits recorded in the COMAV cauliflower collection (*n* = 69 accessions).

Descriptor	Average	SE	CV (%)	Minimum Value	Maximum Value
Stem length below the floral head (cm)	4.11	0.35	72.04	1.43	14.25
Stem diameter (cm)	2.14	0.07	26.95	1.06	3.62
Plant height (cm)	54.23	1.22	19.13	32.00	77.60
Plant width (cm)	81.78	1.99	20.66	40.00	121.20
Head length (cm)	8.20	0.24	24.85	4.20	13.33
Head diameter (cm)	10.91	0.31	24.26	5.10	17.30
Head weight (g)	359.24	15.50	36.61	100.00	723.00
Leaf length (cm)	51.68	1.42	23.26	26.30	82.10
Leaf width (cm)	22.36	0.87	33.02	10.50	49.20
Number of leaves and/or scars	20.01	0.38	16.12	14	26
Petiole length (cm)	1.23	0.07	49.45	1.00	3.20
Petiole width (mm)	2.76	0.11	33.44	0.94	4.80
Petiole thickness (mm)	8.10	0.26	27.68	3.21	13.70
Head stem length (cm)	5.87	0.17	24.79	1.20	8.40
Head stem diameter (cm)	3.24	0.09	24.00	1.20	5.20

**Table 2:** Frequency distribution of qualitative descriptors in the COMAV cauliflower collection (n = 69).

Descriptor	Category	State	Relative Frequency
Plant structure	1	Erect	68%
	2	Semi-erect	32%
	3	Oblique	0%
Leaf colour	1	Yellow-green	0%
	2	Light-green	4%
	3	Green	65%
	4	Dark-green	26%
	5	Purple-green	4%
	6	Purple	0%
	7	Other	0%
Leaf lobing	1	Absent	81%
	9	Present	19%
Leaf blistering	0	None	9%
	3	Low	74%
	5	Intermediate	12%
	7	High	6%
Leaf shape	1	Orbicular	0%
	2	Elliptic	55%
	3	Obovate	10%
	4	Spathulate	12%
	5	Ovate	3%
	6	Lanceolate	16%
	7	Oblong	4%
Leaf division	1	Entire	14%
	2	Sinuate	55%
	3	Lyrate	30%
	4	Lacerate	0%
Leaf contour	0	Absent	2%
	3	Scarce	84%
	5	Intermediate	14%
	7	High	0%
Leaf apex shape	2	Acute	9%
	4	Intermediate	64%
	6	Rounded	2%
	8	Broadly rounded	25%
Petiole section	3	Round	23%
	5	Semi-round	57%
	7	Flat	20%
Petiole colour	1	White	0%
	2	Light-green	84%
	3	Green	9%
	4	Purple	7%
	5	Red	0%
	6	Other	0%
Shape of head longitudinal section	1	Concave	55%
	3	Flat	32%
	5	Spheric	14%
	7	Elliptic	0%
Head formation habit	0	Nonheading	0%
	5	Semi-heading	2%
	7	Heading	98%
Head cover by outer leaves	3	Exposed	57%
	5	Intermediate	41%
	7	Covered	2%
Head surface colour	1	White	55%
	2	Cream	11%
	3	Yellow	2%
	4	Yellow-green	26%
	5	Green	2%
	6	Pink	0%
	7	Green-red	0%
	8	Purple	4%
	9	Red	0%
	10	Orange	0%
	11	Other	0%
Head solidity	3	Low	3%
	5	Intermediate	51%
	7	High	47%
Time between sowing and harvesting	1	<60 days	0%
	2	Between 60 days and 120 days	12%
	3	>120 days	88%
Head size	1	Big	15%
	2	Medium	62%
	3	Small	23%
Inflorescence branching pattern	1	Single flower raceme	0%
	2	Enlarged stem with terminally branched raceme	0%
	3	Loosely branched terminal heads	18%
	4	Terminal head with smaller heads on auxiliary shoots	62%
	5	Compact head of regularly packed subheads	18%
	6	Single compact head of irregularly packed subheads	2%
Ratio head/plant	3	Small	29%
	5	Intermediate	67%
	7	Large	4%
Head stem estimation	3	Short	40%
	5	Intermediate	51%
	7	Long	9%
Head splitting tendency	3	Low	32%
	5	Intermediate	68%
	6	High	0%

**Table 3:** Top 10 traits ranked by contribution to total variance (%).

Trait Name (Acronym)	Contribution to Total Variance (%)
Head surface colour (HCOL)	4.15
Leaf Shape (LSHAPE)	4.02
Shape of Head Longitudinal Section (HLONSEC)	3.05
Leaf Blistering (LEAFBLIST)	2.66
Leaf colour (LEAFCOL)	2.56
Leaf apex shape (LAPEX)	2.28
Petiole Colour (PETCOL)	2.15
Head/plant size ratio (RATHPLAN)	2.08
Head stem estimation (STEMHEST)	1.97
Leaf division (LDIV)	1.91

Table note: Percentages are normalized weighted contributions across the first ten FAMD dimensions; axis weights are proportional to the variance explained by each dimension and rescaled to sum to 1.

**Table 4:** Descriptors and their ID’s and scales used in the phenotyping trial of cauliflower and Romanesco accessions.

Descriptor	Acronym	IPGRI Descriptor ID	Unit/Scores
Number of leaves and/or scars	LSCARS_num	4.2.10	Counted number of leaves and scars
Leaf length	LLENGTH_num	4.2.12	Measured (in cm) largest leave including the petiole
Leaf blade width	LWIDTH_num	4.2.13	Measured (in cm) the largest point of largest leave
Leaf shape	LSHAPE	4.2.16	Leaf blade shape in outline, including lobes
Leaf division	LDIV	4.2.18	1. Entire 2. Sinuate 3. Lyrate 4. Lacerate
Leaf apex shape	LAPEX	4.2.19	2. Acute 4. Intermediate 6. Rounded 8. Broadly rounded
Leaf blistering	LEAFBLIST	4.2.21	0. None 3. Low 5. Intermediate 7. High
Leaf colour	LEAFCOL	4.2.24	1. Yellow-green 2. Light-green 3. Green 4. Dark-green 5. Purple-green 6. Purple 7. Other
Petiole length	PETLENGTH_num	4.2.28	Measured (in cm) where blade intercepts with petiole
Petiole width	PETWIDTH_num	4.2.29	Measured (in mm) in the widest point of the widest leaf, measured in midrib when plant extends to the plant axis
Plant height	PHEIGTH_num	4.2.3	Measured length (in cm) to the extremity of the plant
Petiole thickness	PETTHICK_num	4.2.31	Measured (in mm) in the thickest point of petiole or midrib of largest leaf
Petiole section	PETSEC	4.2.32	3. Round 5. Semi-round 7. Flat
Petiole colour	PETCOL	4.2.33	Petiole and/or midvein colour 1. White 2. Light-green 3. Green 4. Purple 5. Red 6. Other
Head formation habit	HHAB	4.2.34	Observed at harvest 0. Nonheading 5. Semi-heading 7. Heading
Head cover by outer leaves	HCOV	4.2.37	3. Exposed 5. Intermediate 7. Covered
Ratio head/plant	RATHPLAN	4.2.39	3. Small 5. Intermediate 7. Large
Plant width	PDIAM_num	4.2.4	Measured (in cm) width of the plant
Head length	HLENGTH_num	4.2.41	Measured (in cm) median transverse section
Head diameter	HDIAM_num	4.2.42	Measured (in cm) at widest point
Head stem length	STEMHLEN_num	4.2.44	Measured (in cm) stem length in head
Head stem diameter	STEMHDIAM_num	4.2.45	Measured (in cm) stem diameter at head base
Head stem estimation	STEMHEST	4.2.47	Stem length in head estimate
Head splitting tendency	SPLITTEN	4.2.49	3. Low 5. Intermediate 7. High
Stem length below the floral head	SLENGTH_num	4.2.54	Measured (in cm) from cotyledon to the highest point on vegetative or pre-flowering apex
Stem diameter	SDIAM_num	4.2.55	Measured diameter (in cm) of widest point on stem
Head weight	HWEIGTH_num	4.2.6	Weight (in g) of harvested organ
Inflorescence branching pattern	HBRAN	4.2.73	1. Single flower raceme 2. Enlarged stem with terminally branched raceme 3. Loosely branched terminal heads 4. Terminal head with smaller heads on auxiliary shoots 5. Compact head of regularly packed subheads 6. Single compact head of irregularly packed subheads
Shape of head longitudinal section	HLONSEC	4.2.75	1. Concave 3. Flat 5. Spheric 7. Elliptic
Head solidity	HSOL	4.2.77	Flowering head solidity 3. Low (loose) 5. Intermediate 7. High (solid)
Head surface colour	HCOL	4.2.78	Flowering head colour surface 1. White 2. Cream 3. Yellow 4. Yellow-green 5. Green 6. Pink 7. Green-red 8. Purple 9. Red 10. Orange 11. Other
Time between sowing and harvesting	HARVTIME	NA	1. <60 days 2. Between 60 and 120 days 3. >120 days
Head size	HSIZE	NA	G. Big M. Medium P. Small
Leaf contour	LCONTOUR	NA	Leaf blade contour 0. Absent 3. Scarce 5. Intermediate 7. High
Leaf lobing	LEAFLOB	NA	1. Absent 9. Present
Plant structure	PLANTSTR	NA	Foliage habit: 1. Erect 2. Semi-erect 3. Oblique

## Data Availability

The complete phenotypic dataset (21 qualitative + 15 quantitative descriptors for 69 accessions), raw germination counts, R scripts used for FAMD and clustering, and high-resolution figure files will be deposited in the Universitat Politècnica de València institutional repository RiuNet and assigned a DOI immediately after acceptance. Until that time, a read-only link can be provided by the corresponding author upon reasonable request. All other data needed to reproduce the results are contained within the article or its Appendix A.

## Abbreviations

The following abbreviations are used in this manuscript:

COMAV	Instituto Universitario de Conservación y Mejora de la Agro-diversidad Valenciana
CV	Coefficient of Variation
F_1_	First filial-generation hybrid
FAMD	Factorial Analysis of Mixed Data
GVA	*Generalitat Valenciana*
IPGRI	International Plant Genetic Resources Institute
IVIA	*Instituto Valenciano de Investigaciones Agrarias*
MCA	Multiple Correspondence Analysis
PCA	Principal Component Analysis
R	*R* Statistical Computing Environment
SD	Standard Deviation
SE	Standard Error
UPGMA	Unweighted Pair Group Method with Arithmetic Mean
UPV	*Universitat Politècnica de València*

## Appendix A

### Exploratory Dendrogram

For completeness, we also computed a UPGMA dendrogram from Gower distances using all 36 descriptors. The topology broadly agrees with the FAMD-based clustering (e.g., neighbouring provinces tend to group together), and the cophenetic correlation is r = 0.751, indicating that ≈75% of pairwise dissimilarities are preserved (Figure A1). All in-text interpretations are based on the FAMD + clustering results.

## References

[1] Maggioni L., von Bothmer R., Poulsen G., Lipman E. (2018). Domestication, Diversity and Use of *Brassica oleracea* L., Based on Ancient Greek and Latin Texts. Genet. Resour. Crop Evol..

[2] Food and Agriculture Organization of the United Nations FAOSTAT Statistical Database. https://www.fao.org/faostat/en/.

[3] Ministerio de Agricultura Pesca y Alimentación (2023). Anuario de Estadística 2023.

[4] Maggioni L., Ban S.G., Jani S., Jasprica N., Treccarichi S., Išić N., Branca F. (2024). Collecting Mediterranean Wild Species of the Brassica Oleracea Group (Brassica sect. Brassica). Genet. Resour..

[5] Sciortino M., Iapichino G. (2009). Caulifl Ower Hybrids for Spring Production in Southern Mediterranean Area. J. Appl. Hortic..

[6] Bozkurt S., Uygur V., Agca N., Yalcin M. (2011). Yield Responses of Cauliflower (*Brassica oleracea* L. var. *botrytis*) to Different Water and Nitrogen Levels in a Mediterranean Coastal Area. Acta Agric. Scand. B Soil Plant Sci..

[7] Montemurro F., Diacono M., Ciaccia C., Campanelli G., Tittarelli F., Leteo F., Canali S. (2017). Effectiveness of Living Mulch Strategies for Winter Organic Cauliflower (*Brassica oleracea* L. var. *botrytis*) Production in Central and Southern Italy. Renew. Agric. Food Syst..

[8] Witzel K., Kurina A.B., Artemyeva A.M. (2021). Opening the Treasure Chest: The Current Status of Research on Brassica Oleracea and B. Rapa Vegetables from Ex Situ Germplasm Collections. Front. Plant Sci..

[9] Manzanares-Dauleux M.J., Divaret I., Baron F., Thomas G. (2000). Evaluation of French *Brassica oleracea* landraces for resistance to *Plasmodiophora brassicae*. Euphytica.

[10] INRAE—BrasExplor BrasExplor: A Hub for Brassica Genetic Resources. https://brasexplor.hub.inrae.fr/.

[11] Bauer N., Tkalec M., Major N., Talanga Vasari A., Tokić M., Vitko S., Ban D., Ban S.G., Salopek-Sondi B. (2022). Mechanisms of Kale (*Brassica Oleracea* Var. *Acephala*) Tolerance to Individual and Combined Stresses of Drought and Elevated Temperature. Int. J. Mol. Sci..

[12] Gray A.R. (1982). Taxonomy and Evolution of Broccoli (*Brassica oleracea* var. *italica*). Econ. Bot..

[13] Ciancaleoni S., Chiarenza G.L., Raggi L., Branca F., Negri V. (2014). Diversity Characterisation of Broccoli (*Brassica oleracea* L. var. *italica* Plenck) Landraces for Their on-Farm (in Situ) Safeguard and Use in Breeding Programs. Genet. Resour. Crop Evol..

[14] Cuevas H.E., Rosa-Valentin G., Hayes C.M., Rooney W.L., Hoffmann L. (2017). Genomic Characterization of a Core Set of the USDA-NPGS Ethiopian Sorghum Germplasm Collection: Implications for Germplasm Conservation, Evaluation, and Utilization in Crop Improvement. BMC Genom..

[15] Migicovsky Z., Warschefsky E., Klein L.L., Miller A.J. (2019). Using Living Germplasm Collections to Characterize, Improve, and Conserve Woody Perennials. Crop Sci..

[16] Subramanian P., Kim S.-H., Hahn B.-S. (2023). Brassica biodiversity conservation: Prevailing constraints and future avenues for sustainable distribution of plant genetic resources. Front. Plant Sci..

[17] Lei J., Chen G., Chen C., Cao B. (2017). Germplasm Diversity of Chinese Kale in China. Hortic. Plant J..

[18] Govindaraj M., Vetriventhan M., Srinivasan M. (2015). Importance of Genetic Diversity Assessment in Crop Plants and Its Recent Advances: An Overview of Its Analytical Perspectives. Genet. Res. Int..

[19] Swarup S., Cargill E.J., Crosby K., Flagel L., Kniskern J., Glenn K.C. (2021). Genetic Diversity Is Indispensable for Plant Breeding to Improve Crops. Crop Sci..

[20] Razzaq A., Kaur P., Akhter N., Wani S.H., Saleem F. (2021). Next-Generation Breeding Strategies for Climate-Ready Crops. Front. Plant Sci..

[21] Hagos Abraha R., Shaibu A.S., Liang J., Wu J., Lin R., Wang X. (2024). Characterization and Evaluation of the Morphological Attributes of Ethiopian Mustard (*Brassica carinata* A. Braun) Landraces. Euphytica.

[22] Siddiqui M.H., Mohammad F., Khan M.N. (2009). Morphological and Physio-Biochemical Characterization of *Brassica juncea* L. Czern. & Coss. Genotypes under Salt Stress. J. Plant Interact..

[23] Ali F., Ali F., Bibi A., Dessoky E.S., Almowallad S., AlShaqhaa M.A., AL-Balawi S.M., Darwish D.B.E., Allohibi A., Omara M.Y. (2023). Morphological, Biochemical, and Molecular Characterization of Exotic Brassica Germplasm. ACS Omega.

[24] Yousef E.A.A., Müller T., Börner A., Schmid K.J. (2018). Comparative analysis of genetic diversity and differentiation of cauliflower (*Brassica oleracea* var. *botrytis*) accessions from two ex situ genebanks. PLoS ONE.

[25] Kundu P., Murkhejee A., Adhikary A., Ghosal A., Sahu N.C. (2022). Morpho-chemical characterization of broccoli (*Brassica oleracea* var. *italica*). Ann. Plant Soil Res..

[26] Singh S., Bhatia R., Kumar R., Das A., Ghemeray H., Behera T.K., Dey S.S. (2021). Characterization and genetic analysis of OguCMS and doubled haploid based large genetic arsenal of indian cauliflowers (*Brassica oleracea* var. *botrytis* L.) for morphological, reproductive and seed yield traits revealed their breeding potential. Genet. Resour. Crop Evol..

[27] Lotti C., Iovieno P., Centomani I., Marcotrigiano A.R., Fanelli V., Mimiola G., Summo C., Pavan S., Ricciardi L. (2018). Genetic, Bio-Agronomic, and Nutritional Characterization of Kale (*Brassica oleracea* L. var. *acephala*) Diversity in Apulia, Southern Italy. Diversity.

[28] Díez M.J., De la Rosa L., Martín I., Guasch L., Cartea M.E., Mallor C., Casals J., Simó J., Rivera A., Anastasio G. (2018). Plant Genebanks: Present Situation and Proposals for Their Improvement. The Case of the Spanish Network. Front. Plant Sci..

[29] Nguyen G.N., Norton S.L. (2020). Genebank Phenomics: A Strategic Approach to Enhance Value and Utilization of Crop Germplasm. Plants.

[30] El Bakkali A., Essalouh L., Tollon C., Rivallan R., Mournet P., Moukhli A., Zaher H., Mekkaoui A., Hadidou A., Sikaoui L. (2019). Characterization of Worldwide Olive Germplasm Banks of Marrakech (Morocco) and Córdoba (Spain): Towards Management and Use of Olive Germplasm in Breeding Programs. PLoS ONE.

[31] de Oliveira G.L., de Souza A.P., de Oliveira F.A., Zucchi M.I., de Souza L.M., Moura M.F. (2020). Genetic Structure and Molecular Diversity of Brazilian Grapevine Germplasm: Management and Use in Breeding Programs. PLoS ONE.

[32] Jacques A., Duclos D., Danchin-Burge C. (2024). Assessing the Potential of Germplasm Collections for the Management of Genetic Diversity: The Case of the French National Cryobank. Peer Community J..

[33] Centro Nacional de Recursos Fitogenéticos Conjunto de Accesiones de Coliflor. https://www.inia.es/unidades/Institutos%20y%20Centros/CRF/Paginas/Home.aspx.

[34] Fu Y.B. (2024). Will a Plant Germplasm Accession Conserved in a Genebank Change Genetically over Time?. Front. Plant Sci..

[35] Guzzon F., Gianella M., Velazquez Juarez J.A., Sanchez Cano C., Costich D.E. (2021). Seed Longevity of Maize Conserved under Germplasm Bank Conditions for up to 60 Years. Ann. Bot..

[36] Van Treuren R., Bas N., Kodde J., Groot S.P.C., Kik C. (2018). Rapid Loss of Seed Viability in Ex Situ Conserved Wheat and Barley at 4 °C as Compared to −20 °C Storage. Conserv. Physiol..

[37] Rajjou L., Debeaujon I. (2008). Seed Longevity: Survival and Maintenance of High Germination Ability of Dry Seeds. Comptes Rendus Biol..

[38] Walters C., Wheeler L.M., Grotenhuis J.M. (2005). Longevity of Seeds Stored in a Genebank: Species Characteristics. Seed Sci. Res..

[39] Solberg S.Ø., Yndgaard F., Andreasen C., von Bothmer R., Loskutov I.G., Asdal Å. (2020). Long-Term Storage and Longevity of Orthodox Seeds: A Systematic Review. Front. Plant Sci..

[40] Mira S., Estrelles E., González-Benito M.E. (2015). Effect of Water Content and Temperature on Seed Longevity of Seven Brassicaceae Species after 5 Years of Storage. Plant Biol..

[41] Verma D.B., Verma S.S., Tomer U.P.S. (2003). Studies on Seed Quality Parameters in Deteriorating Seeds in Brassica (Brassica campestris).

[42] Sinniah U.R., Ellis R.H., John P. (1998). Irrigation and Seed Quality Development in Rapid-Cycling Brassica: Seed Germination and Longevity. Ann. Bot..

[43] Leeks C.R.F. (2006). Determining Seed Vigour in Selected Bras Sica Species. Master Thesis.

[44] Lasithiotaki L. (2017). Organic Plant Breeding: Seeds for Agro-Biodiversity. Biodiversity.

[45] McMichael B.L., Burke J.J. (1998). Soil Temperature and Root Growth. HortScience.

[46] Finch-Savage W.E., Bassel G.W. (2016). Seed Vigour and Crop Establishment: Extending Performance beyond Adaptation. J. Exp. Bot..

[47] Islam S., Reza M.N., Ahmed S., Samsuzzaman, Cho Y.J., Noh D.H., Chung S.O. (2024). Seedling Growth Stress Quantification Based on Environmental Factors Using Sensor Fusion and Image Processing. Horticulturae.

[48] Oyundelger K., Herklotz V., Harpke D., Oyuntsetseg B., Wesche K., Ritz C.M. (2021). Contrasting Effects of Local Environment and Grazing Pressure on the Genetic Diversity and Structure of *Artemisia frigida*. Conserv. Genet..

[49] FAO (2013). Normas Para Bancos de Germoplasma de Recursos Fitogenéticos para la Alimentación y la Agricultura.

[50] Raza A., Razzaq A., Mehmood S.S., Zou X., Zhang X., Lv Y., Xu J. (2019). Impact of Climate Change on Crops Adaptation and Strategies to Tackle Its Outcome: A Review. Plants.

[51] IBPGR (1990). Descriptors for Brassica and Raphanus.

[52] Rakshita K.N., Singh S., Verma V.K., Sharma B.B., Saini N., Iquebal M.A., Sharma A., Dey S.S., Behera T.K. (2021). Agro-Morphological and Molecular Diversity in Different Maturity Groups of Indian Cauliflower (*Brassica oleracea* var. *Botrytis* L.). PLoS ONE.

[53] Zhu S., Zhang X., Liu Q., Luo T., Tang Z., Zhou Y. (2018). The Genetic Diversity and Relationships of Cauliflower (*Brassica oleracea* var. *botrytis*) Inbred Lines Assessed by Using SSR Markers. PLoS ONE.

[54] Stansell Z., Björkman T. (2020). From Landrace to Modern Hybrid Broccoli: The Genomic and Morphological Domestication Syndrome within a Diverse B. Oleracea Collection. Hortic. Res..

[55] Chen R., Chen K., Yao X., Zhang X., Yang Y., Su X., Lyu M., Wang Q., Zhang G., Wang M. (2024). Genomic Analyses Reveal the Stepwise Domestication and Genetic Mechanism of Curd Biogenesis in Cauliflower. Nat. Genet..

[56] Zhang B., Chen W., Li X., Ren W., Chen L., Han F., Fang Z., Yang L., Zhuang M., Lv H. (2021). Map-Based Cloning and Promoter Variation Analysis of the Lobed Leaf Gene BoLMI1a in Ornamental Kale (*Brassica oleracea* L. var. *acephala*). BMC Plant Biol..

[57] Rousseeuw P.J. (1987). Silhouettes: A Graphical Aid to the Interpretation and Validation of Cluster Analysis. J. Comput. Appl. Math..

[58] Zhao Z., Gu H., Sheng X., Yu H., Wang J., Zhao J., Cao J. (2014). Genetic Diversity and Relationships among Loose-Curd Cauliflower and Related Varieties as Revealed by Microsatellite Markers. Sci. Hortic..

[59] Aleem S., Tahir M., Sharif I., Aleem M., Najeebullah M., Nawaz A., Batool A., Khan M.I., Arshad W. (2021). Principal Component and Cluster Analyses as Tools in the Assessment of Genetic Diversity for Late Season Cauliflower Genotypes. Pak. J. Agric. Res..

[60] Rana N., Sharma A., Rana R.S., Lata H., Bansuli, Thakur A., Singh V., Sood A. (2023). Morphological and Molecular Diversity in Mid-Late and Late Maturity Genotypes of Cauliflower. PLoS ONE.

[61] Thomas C.L., Alcock T.D., Graham N.S., Hayden R., Matterson S., Wilson L., Young S.D., Dupuy L.X., White P.J., Hammond J.P. (2016). Root Morphology and Seed and Leaf Ionomic Traits in a *Brassica napus* L. Diversity Panel Show Wide Phenotypic Variation and Are Characteristic of Crop Habit. BMC Plant Biol..

[62] Chen B., Xu K., Li J., Li F., Qiao J., Li H., Gao G., Yan G., Wu X. (2014). Evaluation of Yield and Agronomic Traits and Their Genetic Variation in 488 Global Collections of *Brassica napus* L.. Genet. Resour. Crop Evol..

[63] Singh J., Sharma A., Sharma P., Kumar N. (2023). Genetic Variability and Association Studies in Mid-Late and Late Group of Cauliflower (*Brassica oleracea* L. var. *botrytis*). Indian J. Plant Genet. Resour..

[64] Kumar M., Sharma S., Kalia P., Saha P. (2011). Genetic variability and character association for yield and quality traits in early maturing indian cauliflowers. Indian J. Hort..

[65] Alemán-Báez J., Qin J., Cai C., Zou C., Bucher J., Paulo M.J., Voorrips R.E., Bonnema G. (2022). Genetic Dissection of Morphological Variation in Rosette Leaves and Leafy Heads in Cabbage (*Brassica oleracea* var. *capitata*). Theor. Appl. Genet..

[66] Lan T.-H., Paterson A.H. (2000). Comparative mapping of quantitative trait loci sculpting the curd of *Brassica oleracea*. Genetics.

[67] Hammond J.P., Broadley M.R., White P.J., King G.J., Bowen H.C., Hayden R., Meacham M.C., Mead A., Overs T., Spracklen W.P. (2009). Shoot Yield Drives Phosphorus Use Efficiency in Brassica Oleracea and Correlates with Root Architecture Traits. J. Exp. Bot..

[68] Zhao Z.Q., Sheng X.G., Yu H.F., Wang J.S., Shen Y.S., Gu H.H. (2020). Identification of QTLs Associated with Curd Architecture in Cauliflower. BMC Plant Biol..

[69] Brendolise C., Espley R.V., Lin-Wang K., Laing W., Peng Y., McGhie T., Dejnoprat S., Tomes S., Hellens R.P., Allan A.C. (2017). Multiple Copies of a Simple MYB-Binding Site Confers Trans-Regulation by Specific Flavonoid-Related R2R3 MYBs in Diverse Species. Front. Plant Sci..

[70] Luo F., Niu G.-B., Zhou Q., Wang L.-J., Bai L.-J., Gao W.-Z. (2023). Transcriptomic and Metabolomic Profiling Reveal the Role of BoMYB2 in Flavor Regulation Mechanism and Coloration in the Postharvest Purple Cauliflower. Postharvest Biol. Technol..

[71] Azpeitia E., Tichtinsky G., Le Masson M., Serrano-Mislata A., Lucas J., Gregis V., Gimenez C., Prunet N., Farcot E., Kater M.M. (2021). Cauliflower Fractal Forms Arise from Perturbations of Floral Gene Networks. Science.

[72] Hulbert S.H., Orton1 T.J. (1984). Genetic and Environmental Effects on Mean Maturity Date and Uniformity in Broccoli. J. Am. Soc. Hortic. Sci..

[73] Takahashi M., Nakano Y., Sasaki H. (2018). Increasing the Yield of Broccoli (*Brassica oleracea* L. var. *italica*) Cultivar ‘Yumehibiki’ during the off-Crop Season by Limiting the Number of Lateral Branches. Hortic. J..

[74] Instituto Valenciano de Investigaciones Agrarias Riegos IVIA. http://riegos.ivia.es/calculo-de-horas-frio.

[75] Baixauli Soria C., Giner Martorell A., Aguilar Olivert J.M., Nájera Juan I. (2017). Aspectos Clave para Diseñar un Programa de Producción en Coliflor.

[76] Federer W.T. (1956). Augmented (or Hoonuiaku) Designs.

[77] Gomes G.P., Baba V.Y., Dos Santos O.P., Sudré C.P., Bento C.D.S., Rodrigues R., Gonçalves L.S.A. (2019). Combinations of Distance Measures and Clustering Algorithms in Pepper Germplasm Characterization. Hortic. Bras..

[78] Wang J.C., Hu J., Guan Y.J., Zhu Y.F. (2013). Effect of the Scale of Quantitative Trait Data on the Representativeness of a Cotton Germplasm Sub-Core Collection. J. Zhejiang Univ. Sci. B.

[79] De Brito M.V., Silva V.B.D.A., Filho C.H.A.M., Ferreira-Gomes R.L., Lopes Â.C.D.A. (2020). Univariate and Multivariate Approaches in the Characterization of Lima Bean Genotypes. Rev. Caatinga.

[80] Valcárcel J.V., Peiró R.M., Pérez-de-Castro A., Díez M.J. (2018). Morphological Characterization of the Cucumber (*Cucumis sativus* L.) Collection of the COMAV’s Genebank. Genet. Resour. Crop Evol..

[81] R Core Team R (2021). A Language and Environment for Statistical Computing.

